# Supplementary figures and images for: Impact of Immunopathy and Coagulopathy on Multi-Organ Failure and Mortality in a Lethal Porcine Model of Controlled and Uncontrolled Hemorrhage
Source: Int J Mol Sci. 2024 Feb 21;25(5):2500. doi: 10.3390/ijms25052500 (PMC10931034; doi:10.3390/ijms25052500)

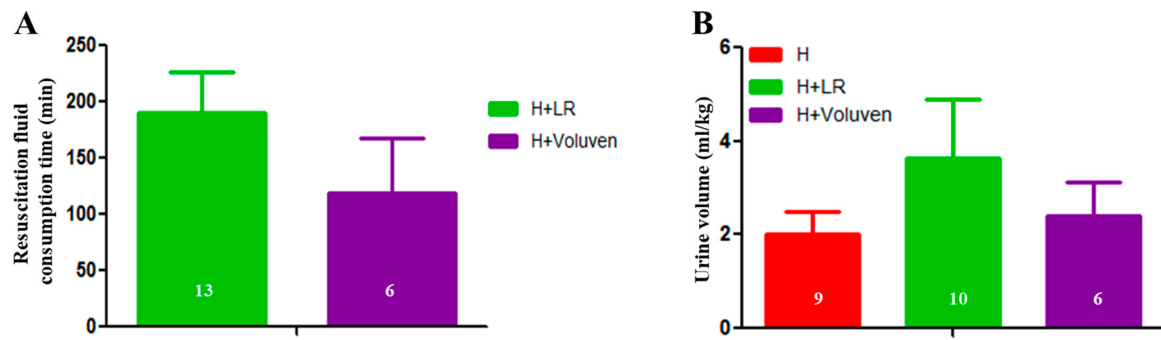

**Supplemental Figure S1.** Fluid resuscitation time and urine output volumes after hemorrhage.

Supplement: Supplementary file 1 [file ijms-25-02500-s001.zip › ijms-2785029-supplementary.pdf]
